# Association between cerebrospinal fluid acidosis and cerebral vasospasm and vacuolation following subarachnoid hemorrhage in a rabbit model

**DOI:** 10.3389/fneur.2025.1649547

**Published:** 2025-09-25

**Authors:** Iskender Samet Daltaban, Sevilay Vural, Mehmet Dumlu Aydin, Mehmet Selim Gel, Ayhan Kanat

**Affiliations:** ^1^Department of Neurosurgery, Alife Hospital, Ankara, Türkiye; ^2^Department of Emergency Medicine, Faculty of Medicine, Yozgat Bozok University, Yozgat, Türkiye; ^3^Department of Neurosurgery, Faculty of Medicine, Atatürk University, Erzurum, Türkiye; ^4^Department of Neurosurgery, Faculty of Medicine, Trabzon University, Trabzon, Türkiye; ^5^Department of Neurosurgery, Faculty of Medicine, Recep Tayyip Erdogan University, Rize, Türkiye

**Keywords:** subarachnoid hemorrhage, vasospasm, cerebrospinal fluid, acidosis, vacuolation

## Abstract

**Introduction:**

Subarachnoid hemorrhage (SAH) is a life-threatening neurological emergency often complicated by delayed cerebral vasospasm, a major cause of morbidity and mortality. Clinical observations indicate that cerebrospinal fluid (CSF) acidosis can develop after aneurysmal SAH and may be associated with delayed cerebral ischaemia.

**Methods:**

We examined the relationship between CSF acidosis, vasospasm severity, and vacuolation in a rabbit model of SAH. Twenty-four hybrid rabbits were randomized into control (*n* = 5), sham-controlled (*n* = 5), and SAH (*n* = 14) groups. SAH was induced by injecting autologous blood into the fourth ventricle; sham-controlled animals received saline. CSF samples were collected on days 1, 7, and 14 for pH analysis, and basilar arteries were harvested on day 14 for determination of a vasospasm index (VSI) and vacuole density (VD). To explore the influence of acidosis severity, SAH animals were stratified into mild (CSF pH ≥ 7.20) and severe (CSF pH < 7.20) acidosis subgroups.

**Results:**

CSF pH was significantly lower in the SAH group than in control or sham-controlled animals (overall mean 7.22 ± 0.03 vs. 7.35 ± 0.02 and 7.31 ± 0.01; *p* < 0.05). While overall VSI did not differ among the groups, animals with severe acidosis displayed a markedly higher VSI than those with mild acidosis (2.83 ± 0.49 vs. 1.60 ± 0.64; *p* = 0.041). VD was elevated in both sham-controlled and SAH groups compared with controls (*p* = 0.009 and *p* = 0.002). Increased vacuolation in sham-controlled animals suggests that surgical manipulation alone can promote vacuole formation. There was a strong inverse correlation between CSF pH and VD (*r* = −0.75, *p* < 0.001), whereas no correlation was found between VSI and VD.

**Discussion:**

CSF acidosis, vacuolation, and vasospasm severity appear to be interconnected factors in the pathophysiology of SAH. CSF acidosis may contribute to vacuolation and, when profound, to vasospasm severity. However, vacuolation and vasospasm represent distinct pathological processes.

## Introduction

Subarachnoid hemorrhage (SAH) is a severe neurological emergency characterized by bleeding into the subarachnoid space, most often due to the rupture of an intracranial aneurysm. Non-traumatic SAH accounts for ~5% of all strokes, with ruptured aneurysms responsible for nearly 85% of cases (1). Despite advances in neurosurgical and critical care management, SAH remains associated with high morbidity and mortality. Survivors frequently develop secondary complications, including hydrocephalus, seizures, delayed cerebral ischemia (DCI), and cerebral vasospasm (VS). Among these, VS is the most common delayed complication and a significant cause of poor neurological outcomes following aneurysmal SAH (aSAH) (2).

Cerebral vasospasm typically occurs between days 3 and 14 after the initial hemorrhage. It involves sustained narrowing of large and medium-sized intracranial arteries, which leads to reduced cerebral perfusion and increases the risk of ischemic injury. The extent of initial hemorrhage is a primary determinant of vasospasm severity. Vasospasm predominantly affects the anterior circulation supplied by the internal carotid arteries, but basilar artery (BA) vasospasm, though less common, carries a particularly poor prognosis due to its potential to cause brainstem ischemia (3). Anatomical factors, including numerous perforating branches and collateral pathways, contribute to the relative rarity of BA vasospasm. Nevertheless, in experimental settings, the BA is frequently studied as a reference vessel due to its accessibility and relatively stable perfusion despite hemodynamic disturbances (4).

The pathophysiology of cerebral vasospasm is complex and multifactorial. Proposed mechanisms include hemoglobin degradation products, endothelial dysfunction, oxidative stress, and inflammation, all of which contribute to vascular smooth muscle contraction and arterial narrowing (5–7). In addition, metabolic acidosis has been documented in the cerebrospinal fluid (CSF) of SAH patients and experimental models, with early-onset acidosis linked to poor prognosis (8, 9). Cohort and translational observations further suggest that CSF acid–base disturbances may be associated with an increased risk of delayed cerebral ischaemia (DCI) and worse neurological outcomes (9, 10). Some authors have described CSF acidosis as one of the most serious complications following SAH (10). However, the specific role of CSF acidosis in the development of vasospasm and structural vascular changes remains unclear.

Another hallmark of vascular injury following SAH is vacuolation within the endothelial and smooth muscle layers of cerebral arteries (11). Prior experimental work in SAH and other cerebrovascular injury models has described vacuolation in these layers; in some reports, higher vacuolation scores coincide with impaired endothelium-dependent vasodilation and greater histopathological injury burden, suggesting potential prognostic relevance rather than a purely incidental finding. In SAH models, impaired endothelium-dependent vasodilation following induction has been demonstrated, with reduced acetylcholine- and ATP-mediated dilatory responses indicating functional impairment that may accompany structural damage (12). Moreover, in experimental models of multiple sclerosis, capillary dilation was found to coincide with localized vacuolation, suggesting that vacuole formation may reflect underlying histopathological burden with potential clinical relevance (8, 13). It is plausible that CSF acidosis contributes to vacuolation by inducing cellular dysfunction or injury; however, this association has not been directly investigated.

This study investigates the effects of CSF acidosis on vasospasm severity and vacuole density in the basilar arteries of rabbits subjected to experimental SAH. By clarifying the relationship between CSF acid-base status and vascular injury, this study seeks to contribute to a better understanding of SAH pathophysiology and to identify potential therapeutic targets.

## Materials and methods

### Study design and overview

This was an exploratory, randomized, controlled animal study designed to examine the association between cerebrospinal fluid (CSF) acidosis and vascular pathology after experimental subarachnoid hemorrhage (SAH). Three groups were studied: control (no intervention), sham (saline injection), and SAH (autologous blood injection). Serial CSF pH measurements were obtained on days 1, 7, and 14, and basilar arteries were harvested on day 14 for histopathological assessment.

### Ethical approval and animal care

All procedures were approved by the Atatürk University Animal Research Ethics Committee (Approval Date: 09.11.2022; Protocol No: 2200369130) and adhered to the NIH Guide for the Care and Use of Laboratory Animals. Animals were monitored daily; predefined humane endpoints (severe distress, persistent pain, moribund state) mandated euthanasia.

### Animals, housing, randomization, and blinding

Twenty-four healthy hybrid rabbits (both sexes, 3–4 months, 2.5–3.0 kg) were acclimatized for 7 days under controlled conditions (12-h light/dark cycle, 21–23 °C, 50–60% humidity) with *ad libitum* access to chow and water. Animals were randomized by a computer-generated sequence to control (*n* = 5), sham (*n* = 5), or SAH (*n* = 14). Three SAH animals died within 72 h after induction (presumed acute intracranial hypertension), leaving *n* = 11 for analysis. Histological and CSF pH assessments were performed by two independent investigators blinded to group allocation.

### Sample size rationale and power considerations

This study was designed as a pilot exploratory investigation, drawing on prior small-animal SAH models and conducted under ethical and resource constraints. Because of these limitations, a formal a priori power analysis was not performed. Group sizes were determined to balance the ethical use of animals with the practical requirements of serial sampling and terminal histology. Consequently, all analyses are interpreted as preliminary and should be viewed with caution. In particular, the small overall cohort and the very limited severe-acidosis subgroup (*n* = 3) substantially reduce statistical power, a limitation that is explicitly acknowledged in the Discussion.

### Anesthesia, peri-operative care, and SAH induction

On day 1, premedication consisted of acepromazine (1 mg/kg IM) and xylazine (5 mg/kg IM), followed by ketamine (35 mg/kg IM) for induction. Anesthesia was maintained with inhaled isoflurane (1–2%) in 100% oxygen, and adequacy was verified by the absence of withdrawal reflexes. Post-procedure, animals received subcutaneous saline and buprenorphine (0.05 mg/kg SC every 12 h for 48 h) and were monitored twice daily for pain, neurological deficits, or systemic complications.

For experimental SAH, rabbits were positioned prone, and the cisterna magna was exposed through a midline suboccipital incision. Using a 27-gauge needle, 0.5 mL of autologous arterial blood (from the central auricular artery) was injected into the fourth ventricle over 60 s, with the needle left *in situ* for 1 min to minimize reflux. Sham animals underwent the same procedure with 0.5 mL of sterile isotonic saline, whereas controls received no surgical intervention. This approach follows established rabbit SAH paradigms and ensures reproducibility of cisterna magna/fourth-ventricle injection–based models.

To minimize variability, all inductions were performed by a single operator using an identical technique. Animals were randomized prior to surgery to avoid allocation bias. Peri-operative conditions were standardized: diet and housing were identical across groups, and no glucose-containing fluids, hyperosmolar therapy, steroids, vasopressors, or vasoactive calcium-channel blockers were administered. Analgesia (buprenorphine) and anesthesia (isoflurane in 100% O_2_) were applied uniformly across all animals.

### Clinical monitoring

Twice-daily welfare checks recorded spontaneous activity, posture/gait, grooming, feeding behavior, righting reflex, and respiratory pattern using a predefined checklist. These observations ensured humane endpoints but were not scored as a formal neurological scale, and no a priori correlation with laboratory or histology variables was planned.

### CSF sampling and pH measurement (equipment/conditions)

CSF (0.3 mL) was aspirated from the cisterna magna under anesthesia on days 1, 7, and 14. Samples were analyzed immediately after collection using a calibrated blood-gas analyser (Radiometer ABL800 FLEX, Copenhagen, Denmark) maintained at 37 °C, per manufacturer recommendations. No storage or cooling was undertaken prior to measurement to avoid temperature-related pH drift. Sampling volume was limited to mitigate CSF depletion.

### Tissue processing and histology

On day 14, animals were euthanised with IV pentobarbital (150 mg/kg). Brains and basilar arteries were harvested and fixed in 10% neutral-buffered formalin for 48 h, paraffin-embedded, sectioned at 5 μm, and stained with haematoxylin-eosin (H&E). Digitized photomicrographs were acquired under standardized illumination and scale-calibrated prior to measurements.

### Image analysis and outcome definitions (consolidated)

All measurements were performed in ImageJ (NIH, USA) on calibrated images by two blinded observers; discrepancies were resolved by consensus.

- **Vasospasm Index (VSI):** on one mid-portion cross-section of the basilar artery, outer radius (R) and luminal radius (r) were measured in two perpendicular axes and averaged. VSI = (R^2^ – r^2^) / r^2^.
- **Vasospasm grading:** no vasospasm (VSI 1–1.5), mild (1.5–2), severe (>2).
- **Vacuolation (Vacuole) Density (VD):** Vacuoles were defined as clear, round/oval spaces within endothelial and/or smooth muscle layers on H&E. Vacuoles were counted in three non-overlapping high-power fields ( × 400) per section and normalized to vessel-wall area, expressed as vacuoles/mm^2^. The mean across fields constituted the per-animal VD.
- **Acidosis subgrouping (SAH animals):** To explore acidosis severity, SAH animals were stratified by overall mean CSF pH into mild acidosis (≥ 7.20) and severe acidosis **(< ** 7.20**)**. The 7.20 threshold reflects both the within-study pH distribution and a clinically/experimentally cited cut-off in prior literature.

### Statistical analysis

Analyses were performed in SPSS v25.0 (IBM, Armonk, NY). Data are reported as mean ± SD. Given small samples and non-normality, Kruskal–Wallis tests assessed overall group differences, with Mann–Whitney U tests for pairwise comparisons where appropriate. Associations among CSF pH, VSI, and VD were examined using Spearman's rank correlation. Two-sided *p* < 0.05 denoted statistical significance. In line with the exploratory pilot design, no formal multiplicity adjustment was applied; exact *p*-values are reported and interpreted cautiously. Predefined outcomes and consolidated definitions (VSI, VD, acidosis subgroups) are presented above to enhance reproducibility.

## Results

A total of 24 rabbits were initially included in the study. During the experimental period, three animals assigned to the subarachnoid hemorrhage (SAH) group died within the first 72 h following the blood injection procedure. These deaths were attributed to complications associated with acute intracranial hypertension. Consequently, the final sample size for analysis consisted of 21 rabbits: five in the control group, five in the sham group, and 11 in the SAH group.

### CSF pH measurements

Cerebrospinal fluid (CSF) pH levels were measured on days 1, 7, and 14 following the intervention. The SAH group demonstrated significantly lower CSF pH values compared to both control and sham groups at all time points. The overall mean CSF pH values, calculated as the average of the three sequential measurements, were 7.35 ± 0.02 in the control group, 7.31 ± 0.01 in the sham group, and 7.22 ± 0.03 in the SAH group. Statistical analysis revealed a significant difference in overall CSF pH levels among the groups (Kruskal–Wallis test, *p* < 0.001). Pairwise comparisons confirmed significant differences between control and sham groups (*p* = 0.047), control and SAH groups (*p* = 0.002), and sham and SAH groups (*p* = 0.002; Mann–Whitney U test). Because this was an exploratory pilot study with small samples, no formal multiplicity adjustments (e.g., Bonferroni/Holm) were applied; exact two-sided *p*-values are reported descriptively.

**Figure 1:**
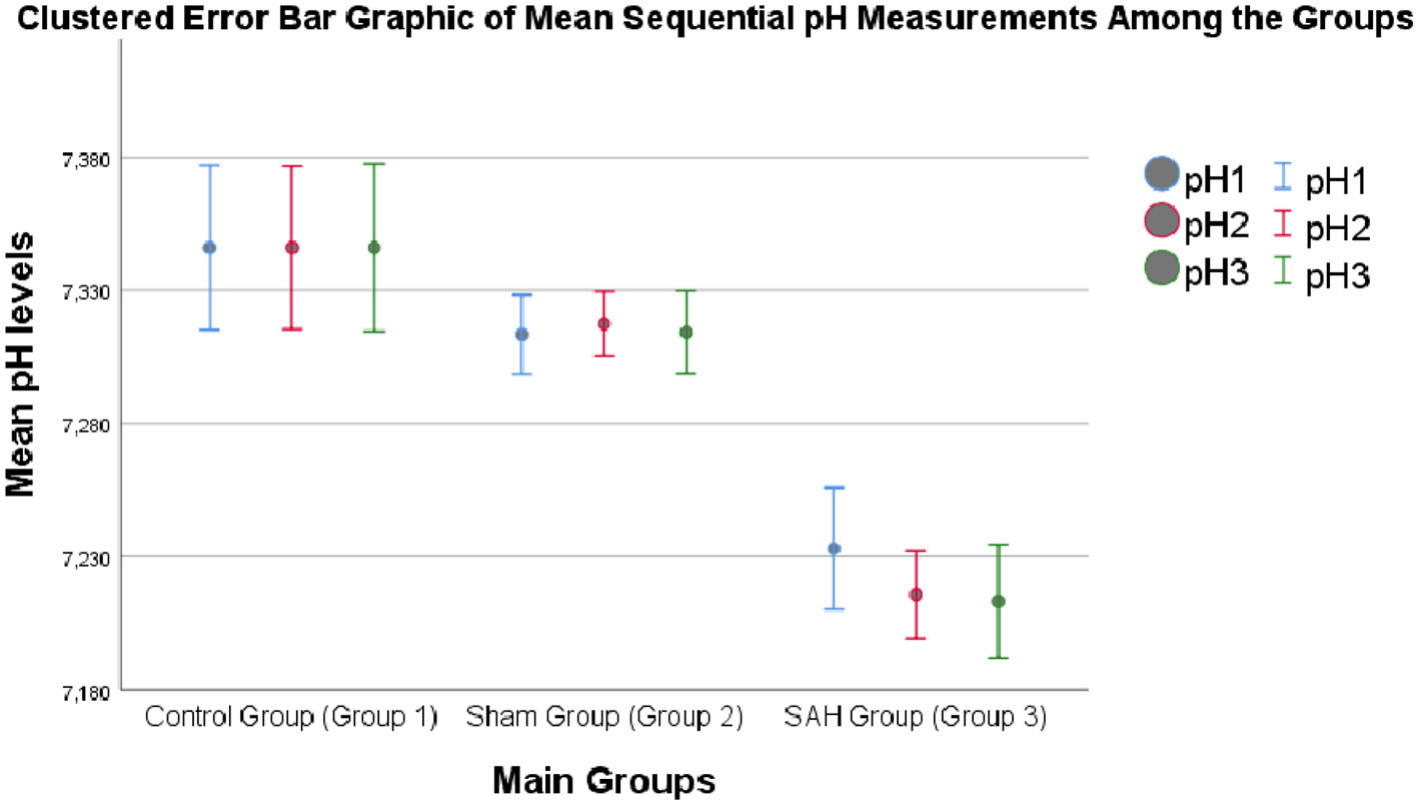
The clustered error bar graphic of the sequential pH measurements of the study groups during the experiment (Day 1, 7, and 14).

As shown in Table 1 and Figure 1, at each time point (days 1, 7, and 14) CSF pH remained lower in SAH vs. both control and sham groups, with a small transient reduction in the sham group vs. controls at days 1 and 7 that resolved by day 14. Similarly, at individual time points (days 1, 7, and 14), CSF pH was consistently lower in the SAH group compared to both control and sham animals. Additionally, on days 1 and 7, the sham group exhibited slightly lower CSF pH values compared to the control group (*p* = 0.047), although by day 14, no significant difference was detected between these two groups (*p* = 0.76; Table 1, Figure 1).

**Table 1:** The sequential and overall pH, vasospasm index (VSI) and vacuole density (VD) measurements of the study groups during the experiment (Day 1, 7, and 14).

**Groups**	**pH (day 1)**	**pH (day 7)**	**pH (day 14)**	**Overall pH**	**VSI**	**VD**
Control (*n* = 5)	7.35 ± 0.02	7.35 ± 0.02	7.35 ± 0.03	7.35 ± 0.02	1.16 ± 0.17	0.021 ± 0.017
Sham (*n* = 5)	7.31 ± 0.01	7.32 ± 0.01	7.31 ± 0.01	7.31 ± 0.01	1.32 ± 0.39	0.163 ± 0.060
SAH (*n* = 11)	7.23 ± 0.03	7.22 ± 0.02	7.21 ± 0.03	7.22 ± 0.03	1.94 ± 0.81	0.22 ± 0.073
^*^p	0.001	< 0.001	< 0.001	< 0.001	0.157	0.002
^†^Control *vs*. Sham	0.047	0.047	0.76	0.047	0.251	0.009
^†^Control *vs*. SAH	0.002	0.002	0.002	0.002	0.112	0.002
^†^Sham *vs*. SAH	0.004	0.002	0.002	0.002	0.193	0.157

All parameters were presented as mean ± standard deviation. SAH, subarachnoid hemorrhage; VSI, vasospasm index; VD, vacuole density. ^*^Kruskal–Wallis test, ^†^Mann Whitney u test.

### Vasospasm index (VSI)

The vasospasm index (VSI) was measured in basilar arteries on day 14. The mean VSI values were 1.16 ± 0.17 in the control group, 1.32 ± 0.39 in the sham group, and 1.94 ± 0.81 in the SAH group. Although VSI tended to be higher in the SAH group, these differences did not reach statistical significance when comparing all three groups (Kruskal–Wallis test, *p* = 0.157). According to the VSI grading scale, mean values in all groups fell within the range interpreted as no vasospasm to mild vasospasm. Notably, none of the groups demonstrated an average VSI exceeding the threshold for severe vasospasm (VSI > 2).

### Vacuole density (VD)

Vacuole density (VD), defined as the number of vacuoles per square millimeter of vessel wall, was markedly elevated in both the sham and SAH groups compared with controls. The mean VD values were 0.021 ± 0.017 vacuoles/mm^2^ in the control group, 0.163 ± 0.060 vacuoles/mm^2^ in the sham group, and 0.220 ± 0.073 vacuoles/mm^2^ in the SAH group. Overall group differences were significant (Kruskal–Wallis test, *p* = 0.002, η^2^ = 0.42, indicating a large effect). Pairwise comparisons confirmed that both the sham (*p* = 0.009, Cohen's *d* = 2.98) and SAH (*p* = 0.002, Cohen's *d* = 3.43) groups had substantially higher VD than controls, whereas the sham–SAH difference did not reach statistical significance (*p* = 0.157, Cohen's *d* = 0.86). Beyond statistical significance, the effect sizes suggest biological relevance: VD was ~8-fold higher in sham vs. controls (0.163 ± 0.060 vs. 0.021 ± 0.017 vacuoles/mm^2^) and nearly 10-fold higher in SAH vs. controls (0.220 ± 0.073 vs. 0.021 ± 0.017 vacuoles/mm^2^; Table 1).

### Correlation analysis

Correlation analysis revealed a strong negative association between overall CSF pH and vacuole density (Spearman's rank-order correlation coefficient, *r* = −0.75, *p* < 0.001), indicating that lower CSF pH was associated with greater vacuole formation in the basilar artery wall. In contrast, no significant correlation was found between VSI and VD (*p* = 0.244), suggesting that vacuolation and vasospasm may occur through independent mechanisms in this model. The magnitude of the association (|r| = 0.75) indicates a strong relationship between lower CSF pH and greater vacuole burden; nonetheless, the observational design precludes causal inference.

### Subgroup analysis of SAH animals

Within the SAH group, animals were stratified according to the severity of CSF acidosis: mild acidosis (CSF pH ≥ 7.20, *n* = 8) and severe acidosis (CSF pH < 7.20, *n* = 3). As shown in Table 2, the mean VSI was 2.83 ± 0.49 in the severe subgroup compared with 1.60 ± 0.64 in the mild subgroup, representing a 1.23-unit absolute increase and an ~77% relative rise. Despite the limited statistical power due to the small subgroup size, this difference reached significance (Mann–Whitney U test, *p* = 0.041) and yielded a large effect size (Cohen's *d* = 2.2), underscoring potential biological relevance of profound acidosis. Furthermore, VSI values in the severe acidosis subgroup were significantly higher than those observed in both the control and sham groups (Kruskal–Wallis test, *p* = 0.027; Mann–Whitney U test, *p* = 0.025 for both comparisons). By contrast, vacuole density did not differ between mild and severe acidosis subgroups (0.217 ± 0.082 vs. 0.215 ± 0.057 vacuoles/mm^2^, *p* = 0.683; Table 2), suggesting that the effect of acidosis severity was more pronounced on vasospasm indices than on structural vacuolation.

**Table 2:** The vasospasm index (VSI) and vacuole density (VD) measurements of the subarachnoid hemorrhage (SAH) subgroups.

**SAH subgroups**	**VSI**	**VD**
Mild acidosis (*n* = 8)	1.60 ± 0.64	0.217 ± 0.082
Severe acidosis (*n* = 3)	2.83 ± 0.49	0.215 ± 0.057
^†^ *p*	**0.041**	0.683

All parameters were presented as mean ± standard deviation. SAH, subarachnoid hemorrhage; VSI, vasospasm index; VD, vacuole density. ^†^Mann Whitney u test. Bold characters indicate statistical significance.

### Macroscopic and histopathological findings

**Figure 2:**
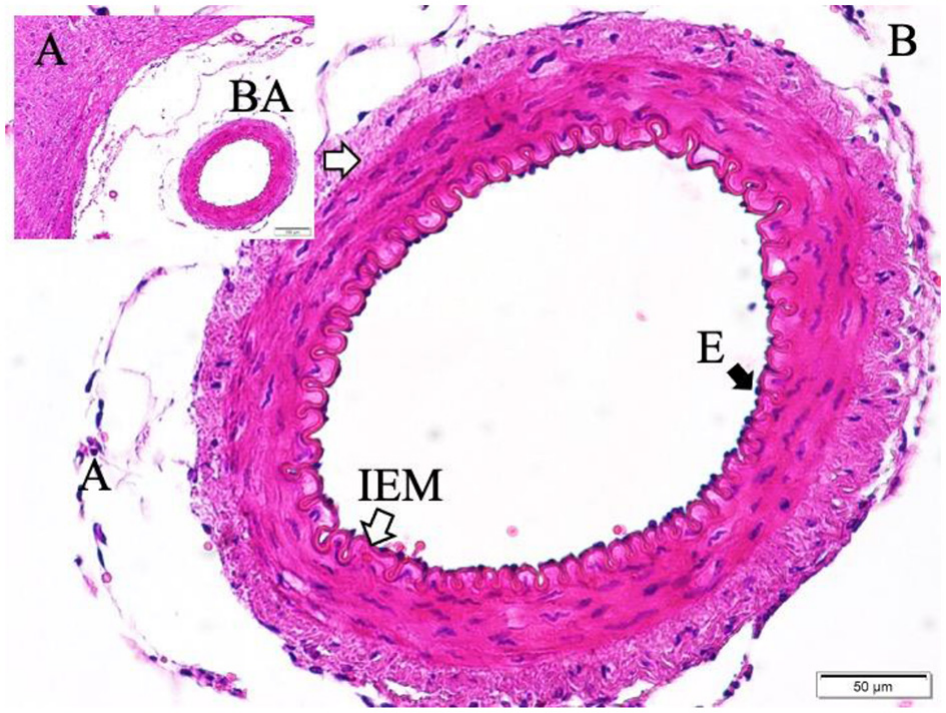
Basilar artery (BA) in basilar sulcus at the anterior surface of pons (LM, H&E, x4/A) and a magnified view with inner elastic membrane (IEM), endothelial cells (E), and normal muscles in the basilar artery wall in a control subject (LM, H&E, x20/B).

**Figure 3:**
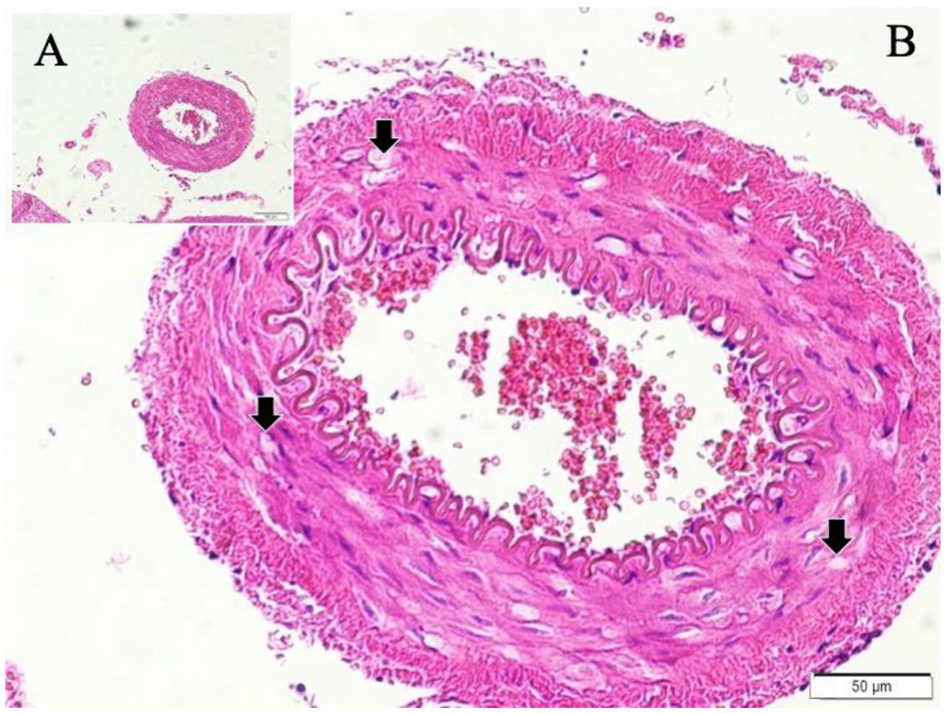
Basilar artery in bloody basilar sulcus at the anterior surface of pons (LM, H&E, x4/A) and a magnified view of basilar artery with periadventitial blood cells with severe convoluted inner elastic membrane (IEM), prominent ischemic endothelial cells (E), contracted muscles and fluid some extent filled vacuoles (Arrows) in the basilar artery wall in a subarachnoid hemorrhage subject with mild acidosis (LM, H&E, x20/B).

**Figure 4:**
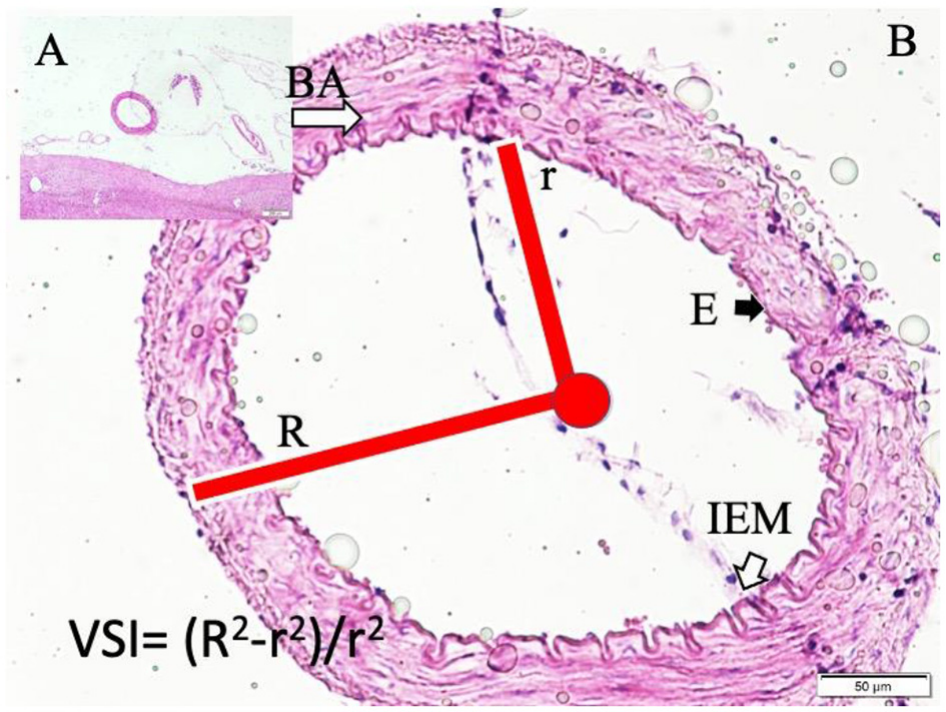
Basilar artery (BA) in the basilar sulcus at the anterior surface of pons (LM, H&E, x4/A) and a magnified view with moderate convoluted inner elastic membrane (IEM), ischemic endothelial cells (E), and minimally contracted muscles in the basilar artery wall in a sham subject (LM, H&E, x20/B). VSI estimation parameters and method are also shown.

**Figure 5:**
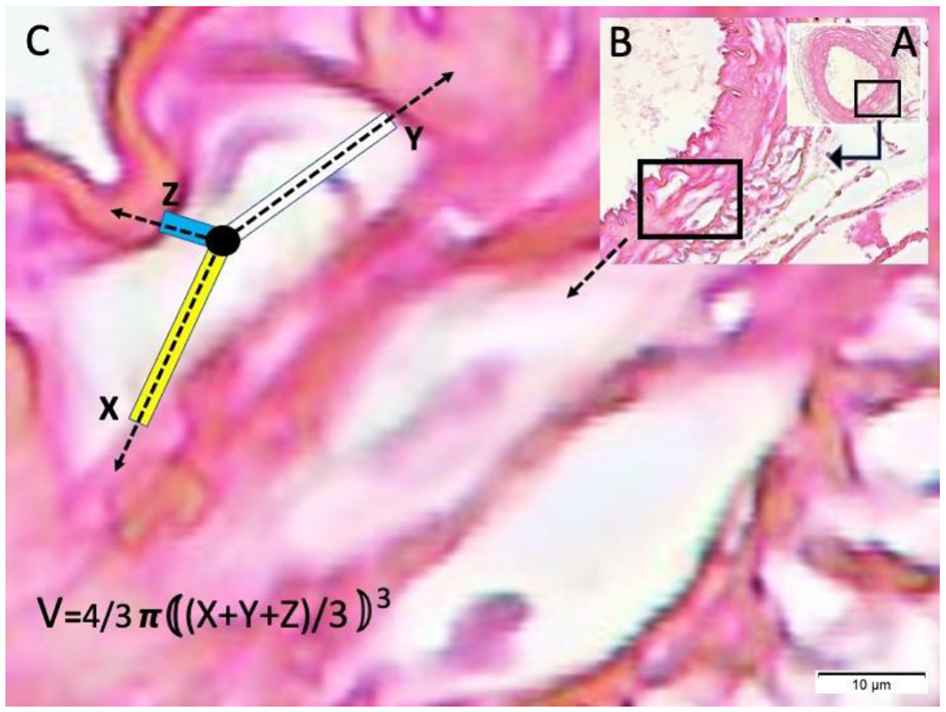
Basilar artery in bloody basilar sulcus at the anterior surface of pons (LM, H&E, x4/A) and a magnified view of basilar artery with periadventitial edema, severe convoluted inner elastic membrane, prominent ischemic endothelial cells, contracted muscles and extended water filled vacuoles in edematous vessel wall (LM, H&E, x10/B) and vacuole count estimation parameters and method is shown in a subarachnoid hemorrhage subject with severe acidosis (LM, H&E, x100/B).

Gross postmortem examination of the brains in the SAH group revealed multiple pathological changes, including visible subarachnoid hemorrhage, cerebral swelling, increased intracranial pressure, thickening of the arachnoid membrane, loss of Sylvian fissures and sulcal structures, basilar artery thrombosis, and clotted blood compressing the basilar artery. Necropsy further confirmed subarachnoid clotting within the basal cisterns. Representative images of these findings are provided in Figures 2–5. In contrast, sham animals did not exhibit subarachnoid clots, although mild meningeal thickening was observed in some cases.

Histopathological evaluation of the basilar arteries confirmed the presence of luminal narrowing, convolution of the internal elastic membrane, intimal edema, endothelial cell swelling, muscular layer swelling, endothelial desquamation, and evidence of apoptosis in both endothelial and smooth muscle cells in SAH animals. In 20% of SAH animals, the microarterioles supplying the basilar artery were absent on histological sections, potentially indicating thrombosis or obliteration of these vessels.

Representative histological images demonstrate the differences among the study groups. In control animals, the basilar artery wall architecture was normal, with intact endothelium and elastic membranes (Figure 2). In sham animals, moderate convolution of the internal elastic membrane and ischemic changes in endothelial cells were observed (Figure 3).

In SAH animals with mild acidosis, more pronounced changes were evident, including periadventitial blood infiltration, convoluted elastic membranes, ischemic endothelial cells, contracted smooth muscle cells, and vacuole formation within the vessel Wall (Figure 4).

SAH animals with severe acidosis exhibited extensive periadventitial edema, severe convolution of the internal elastic membrane, contracted muscular layers, and large vacuoles filled with fluid (Figure 5).

## Discussion

Our findings indicate a strong association between cerebrospinal fluid acidosis and vacuole formation in the basilar artery wall, as well as greater vasospasm severity in the presence of profound acidosis. Nevertheless, because this was an observational animal study with small subgroup sizes, these results cannot establish causality and should be interpreted with caution. Importantly, we did not observe a direct correlation between vacuolation and vasospasm severity, suggesting that these pathological processes may arise through partly independent mechanisms. To our knowledge, this is the first experimental SAH study to jointly examine CSF acidosis, vacuole formation, and vasospasm, thereby providing preliminary insights while underscoring the need for further validation in larger cohorts and complementary models.

Although acid-base disturbances, particularly acidosis, are frequently encountered in critically ill patients, their direct impact on central nervous system (CNS) pathology is less well understood (8, 14). In clinical settings, systemic blood pH is typically emphasized, whereas CSF pH is often overlooked. However, just as systemic acidosis can cause cellular and tissue injury, CSF acidosis may exert similarly harmful effects within the CNS. Jalalvand et al. demonstrated that normal neuronal transmission occurs at a physiological pH of 7.4 but is significantly impaired under both acidotic and alkalotic conditions, resulting in disrupted neuronal function (15).

In our experimental model, SAH subjects exhibited significantly lower CSF pH levels compared to both control and sham animals. This sustained CSF acidosis aligns with previous findings by Kocak et al., who demonstrated severe spinal cord injury in the presence of acidotic CSF (8). However, clinical studies show conflicting results. Langer et al. reported localized CSF acidosis in SAH patients but found no significant difference in overall CSF pH compared to controls (16). Conversely, Suzuki et al. observed that higher CSF pH and lower CSF pCO_2_ levels were associated with delayed cerebral ischemia (DCI) after SAH (17). These discrepancies may be attributed to differences in patient populations, the timing of CSF sampling, and medical interventions, such as hyperventilation or aggressive CSF drainage strategies. Inadequate CSF drainage may impede the clearance of clots and acidic metabolites, potentially contributing to persistent acidosis and impaired CSF gas exchange, which in turn may promote vasospasm and DCI (18).

Another key observation of this study is the morphological alteration of cerebral arteries, which is considered an important component in the pathogenesis of vasospasm following SAH. Structural changes in the vascular intima, media, and adventitia, including endothelial distortion, vacuole formation, tight junction disruption, and widening of interendothelial spaces, have been reported in previous studies and are considered markers of endothelial dysfunction and cellular stress during the acute and subacute phases of SAH (19). While the precise role of vacuolation in vasospasm remains unclear, it is plausible that these changes may alter vascular compliance or contribute to sustained endothelial dysfunction.

In our study, vacuole density was significantly increased in SAH animals compared to controls, regardless of acidosis severity. Notably, sham-operated animals also demonstrated increased vacuolation, suggesting that factors beyond SAH-induced acidosis, such as surgical manipulation, transient increases in intracranial pressure, or aseptic inflammation, may induce vacuole formation. Similar vascular vacuolation/vasodilation has been observed in other CNS conditions, including traumatic brain injury and Alzheimer's disease, where it is regarded as a general marker of cellular stress or injury (20).

A study by Yan et al. demonstrated vacuole accumulation in cerebral arterioles as early as 8 weeks after experimental brain microinjury, with subsequent neuronal apoptosis and vasospasm (21). While our findings partially align with theirs, we did not observe a correlation between vacuole density and vasospasm index (VSI). This difference may be attributable to the shorter time frame of our experimental model (14 days) vs. the chronic nature of their study (8 weeks). It is possible that vacuole formation contributes to long-term vascular remodeling and chronic vasospasm, effects not captured within the acute-to-subacute period studied here.

Our results suggest that vacuolation and vasospasm may arise through distinct pathophysiological pathways. CSF acidosis appears to be a shared contributing factor, yet vacuole formation likely reflects endothelial or smooth muscle cell injury, whereas vasospasm involves active vascular smooth muscle contraction triggered by biochemical stimuli such as hemoglobin breakdown products or inflammatory mediators (4). The absence of a direct correlation between these processes highlights the complexity of SAH pathophysiology. The rabbit SAH model reproduces several salient features of post-haemorrhagic pathology, including exposure of large arteries to blood products, delayed vasospasm, and acid–base disturbances, yet species-specific differences in vascular anatomy, vasa vasorum distribution, CSF dynamics, and neurovascular coupling limit the extent to which these findings can be extrapolated to humans. Clinical observations in aneurysmal SAH report localized CSF acid, base disturbances that conceptually align with our experimental data. Nevertheless, these results should be considered preliminary and hypothesis-generating, and controlled human studies will be required to determine whether comparable acid–base dynamics contribute to vacuolation or vasospasm in patients.

Finally, the observation that severe CSF acidosis correlates with vasospasm severity highlights the potential therapeutic relevance of addressing acid-base imbalances following SAH. Early intervention to correct CSF acidosis may mitigate vascular injury and reduce the risk of vasospasm. However, further experimental studies are needed to assess whether CSF pH normalization improves vascular outcomes.

This exploratory study has several important limitations. First, the overall cohort was small, and the severe-acidosis subgroup included only three animals, which markedly limited statistical power; three early deaths in the SAH group further reduced the number of analyzable cases. Second, we examined a single species and focused on one vascular territory (the basilar artery) at a single terminal histological time point, which restricts generalizability across cerebrovascular regions and time scales. Third, systemic acid–base parameters were not measured, preventing us from distinguishing localized CSF acidosis from systemic disturbances. Fourth, CSF pH was not experimentally manipulated (e.g., buffering or drainage), limiting causal inference. Fifth, consistent with the pilot design, no formal multiplicity adjustments were performed; exact *p*-values are reported descriptively, which may increase the risk of type I error. Sixth, early imaging-based grading of hemorrhage burden (analogous to human Fisher scores) was not obtained, and reliance on procedural standardization and terminal necropsy therefore limits precision in assessing initial severity. Finally, systemic biochemical variables such as serum electrolytes (e.g., sodium) and glucose were not systematically recorded, precluding adjustment for these potential modifiers of vasospasm risk.

Future work should include larger sample sizes, extended follow-up to evaluate chronic vascular remodeling, functional outcome assessments, and direct manipulation of CSF pH to clarify causal relationships. Although our findings suggest that CSF acid–base disturbances may be associated with vacuolation and, when severe, with heightened vasospasm severity, these results are derived from an experimental rabbit model and should not be directly extrapolated to clinical practice. Whether CSF pH monitoring or targeted correction strategies (e.g., drainage techniques or buffering interventions) can improve vascular or neurological outcomes remains uncertain and requires rigorous, prospectively designed clinical studies with adequate statistical power.

If validated in patients, serial CSF pH monitoring, where feasible via existing external ventricular drains, could complement standard tools such as clinical examination, transcranial Doppler, and vascular imaging to identify patients at greater biological risk. pH-targeted strategies warrant systematic evaluation in controlled settings, including: (i) optimized CSF drainage protocols to facilitate removal of acidic metabolites, (ii) carefully titrated buffering or irrigation approaches to normalize CSF acid–base balance, and (iii) systemic management strategies aimed at maintaining normocapnia and metabolic homeostasis. These approaches remain hypothesis-generating and will require rigorous safety, feasibility, and efficacy testing in appropriately powered translational and clinical studies.

## Conclusion

Our data indicate that lower cerebrospinal fluid (CSF) pH is associated with greater vacuolation of the basilar artery wall and, when acidosis is profound, with higher vasospasm severity in a rabbit SAH model. These associations suggest that CSF acid–base status may represent a modifiable factor; however, in the absence of direct interventional experiments, any reference to “pH-targeted therapy” should be regarded as a hypothesis-generating concept rather than a clinical implication.

This pilot study was limited by small group sizes, single species, and a short observation window. Therefore, the findings should be interpreted cautiously. Nevertheless, the concurrent analysis of CSF pH, vasospasm, and vacuolation provides novel insights and underscores the need for larger, multi-species, and interventional studies to clarify causality and translational relevance.

## Data Availability

The raw data supporting the conclusions of this article will be made available by the authors, without undue reservation.
